# A novel brain tumour model in zebrafish reveals the role of YAP activation in MAPK- and PI3K-induced malignant growth

**DOI:** 10.1242/dmm.026500

**Published:** 2017-01-01

**Authors:** Marie Mayrhofer, Victor Gourain, Markus Reischl, Pierre Affaticati, Arnim Jenett, Jean-Stephane Joly, Matteo Benelli, Francesca Demichelis, Pietro Luigi Poliani, Dirk Sieger, Marina Mione

**Affiliations:** 1 Institute for Toxicology and Genetics, Hermann von Helmholtz Platz 1, Eggenstein-Leopoldshafen 76344, Germany; 2 Institute for Applied Informatics at Karlsruhe Institute of Technology, Hermann von Helmholtz Platz 1, Eggenstein-Leopoldshafen 76344, Germany; 3 Tefor Core Facility, Paris-Saclay Institute of Neuroscience, CNRS, Université Paris-Saclay, Gif-sur-Ivette 91190, France; 4 Centre for Integrative Biology, University of Trento, Via Sommarive 9, Trento 38123, Italy; 5 Department of Molecular and Translational Medicine, Pathology Unit, University of Brescia School of Medicine, Spedali Civili Brescia, Brescia 25123, Italy; 6 Centre for Neuroregeneration, The University of Edinburgh, The Chancellor's Building, 49 Little France Crescent, Edinburgh EH16 4SB, UK

**Keywords:** Glioma, YAP, RAS, Zebrafish, Heterotopia

## Abstract

Somatic mutations activating MAPK and PI3K signalling play a pivotal role in both tumours and brain developmental disorders. We developed a zebrafish model of brain tumours based on somatic expression of oncogenes that activate MAPK and PI3K signalling in neural progenitor cells and found that HRAS^V12^ was the most effective in inducing both heterotopia and invasive tumours. Tumours, but not heterotopias, require persistent activation of phospho (p)-ERK and express a gene signature similar to the mesenchymal glioblastoma subtype, with a strong YAP component. Application of an eight-gene signature to human brain tumours establishes that YAP activation distinguishes between mesenchymal glioblastoma and low grade glioma in a wide The Cancer Genome Atlas (TCGA) sample set including gliomas and glioblastomas (GBMs). This suggests that the activation of YAP might be an important event in brain tumour development, promoting malignant versus benign brain lesions. Indeed, co-expression of dominant-active YAP (YAP^S5A^) and HRAS^V12^ abolishes the development of heterotopias and leads to the sole development of aggressive tumours. Thus, we have developed a model proving that neurodevelopmental disorders and brain tumours might originate from the same activation of oncogenes through somatic mutations, and established that YAP activation is a hallmark of malignant brain tumours.

## Abstract (precis)

**Editors' choice:** A new brain tumour model in zebrafish allows simultaneous analysis of malignant tumours and heterotopias, suggesting that both lesions originate from similar events, with Yap1 as a driving force in tumour development.

## INTRODUCTION

Disorders of brain growth are known to cause a wide range of physiological and pathological symptoms such as intractable epilepsy, intellectual disability, autism, and cognitive and motor impairment (Aronica and Crino, 2014; Courchesne et al., 2001; Hevner, 2015). Their causes are diverse and comprise: (1) focal lesions characterised by abnormal location of otherwise normally differentiated neural cells (Thom et al., 2004), (2) ‘tuberous’ formation and similar disorders with abnormally large neurons and/or other cell types (Blümcke et al., 2011; Crino, 2013) and (3) brain overgrowth syndromes leading to diffuse megalencephaly or malformations (Winden et al., 2015), which are mostly limited to developmental stages. By contrast, paediatric and adult primary malignant brain tumours are characterised by the sustained proliferation of poorly differentiated or abnormal neural cells. Both brain growth disorders and tumours might initially appear with similar symptoms and share a similar origin from somatic mutations (Blümcke et al., 2011), which is confirmed by co-occurrence of both disorders in the same individuals (Johansson et al., 2015). For example, coexistence of developmental disorders and brain tumours is commonly observed in neurofibromatosis type 1 (NF1) (Reuss and von Deimling, 2009), a genetic disorder that afflicts 1 in ∼3000 newborns (Evans et al., 2010). NF1 is caused by loss-of-function mutations in the tumour suppressor gene *NF1*, which encodes neurofibromin 1, a negative regulator of the proto-oncogene *RAS* (Ballester et al., 1990; Cichowski and Jacks, 2001) and in 50% of cases occurs as the result of *de novo* somatic mutations. Between 5% and 10% of individuals with tuberous sclerosis (caused by mutations in *TSC1* or *TSC2*, OMIN 191100) develop slowly growing subependymal giant cell astrocytomas (Grajkowska et al., 2010) and in 50% of individuals with focal cortical dysplasia type 3b cortical disorganisation masks slowly developing brain tumours (Blümcke et al., 2011; Palmini et al., 2013; Santos et al., 2014).

Conversely, for most developmental disorders of the brain it is currently unknown whether focal or diffuse growth disorders might progress to tumours as a result of additional mutations or epigenetic events. Although the molecular pathogeneses of these disorders are currently unknown, for most of them genetic studies show that activation of MAPK, PI3K or mTOR signals resulting from *de novo* somatic mutations or inherited germline mutations might be causative (for review see Aronica and Crino, 2014; Barkovich et al., 2012; Dyment et al., 2013; Hevner, 2015). These pathways are also altered in gliomas, as leading mutations in high-grade gliomas include EGFR amplification (in 27-36% of cases; Ohgaki and Kleihues, 2007) or mutations (18-31% of cases; Liu et al., 2005), deletion of PTEN, the inhibitor of AKT and mTOR (15-40% of cases; Tohma et al., 1998) and inactivation of NF1, a RAS inhibitor (18% of cases; The Cancer Genome Atlas Research Network, 2008). Whereas only few gliomas contain mutation in *RAS* itself, the leading mutations reported above affect its activity in nearly all glioma cases (Jones et al., 2012; Patil et al., 2013). By contrast, activation of RAS and/or RAF is the molecular hallmark of pilocytic astrocytoma (a grade I astrocytoma, which rarely progresses to higher grade; Jones et al., 2008).

Although separate models for developmental disorders and brain tumour diseases exist (Fomchenko and Holland, 2006; Stylli et al., 2015; Wong and Roper, 2015), models of progressive brain developmental disorders with spontaneous cancerous development are lacking. Such models might allow the study of the molecular events leading to tumour development starting from benign developmental lesions, the identification of the mechanisms of tumour suppression in those cases that do not progress, and the development of preventive therapies. These models could also be instrumental in understanding the progression from benign to malignant brain tumours, such as glioblastoma (GBM), which occurs in ∼50% of individuals diagnosed with grade II and grade III gliomas (Chaichana et al., 2010; Ho et al., 2016, Louis et al., 2016).

Glioma progression has been linked to a number of pathway alterations including EGFR/MAPK/PTEN and p53 signalling (Ohgaki and Kleihues, 2009). Additionally, the transcriptional co-activators YAP and TAZ of the Salvador–Warts–Hippo pathway have been linked to glioma progression and poor patient survival (Bhat et al., 2011; Orr et al., 2011). An increase in YAP and TAZ activity has been documented in high grade gliomas, although they are significantly less active in low grade gliomas (Orr et al., 2011). In cell culture experiments YAP and TAZ promote glioma cell proliferation, invasion and resistance to apoptosis (Bhat et al., 2011; Orr et al., 2011). However, their impact on the progression of low grade gliomas is still poorly documented.

In this study we have generated a zebrafish model of focal brain growth disorders through the expression of different oncogenes in neural cells during development. These focal growths are of two types; either they result in dislocation of neural cells (or duplication of neural structures) without further growth, malformations defined as ‘heterotopias’ or in malignant brain tumours. Thus, this model provides novel insights into the relation between benign lesions and aggressive tumours as it shows that: (1) RAS/MAPK signalling can induce both heterotopia (non-cancerous benign lesions) and aggressive brain tumours; (2) aggressive tumours have a mesenchymal GBM signature; (3) activation of YAP signalling distinguishes aggressive tumours from heterotopia; and (4) forcing the activation of YAP signalling at earlier stages promotes aggressive tumours at the expenses of heterotopia. These data indicate a central role for YAP activation in the progression of benign growth disorders to aggressive tumours and suggest the possibility of preventing it by specific inhibitors.

## RESULTS

### Activation of the EGFR/RAS/ERK/AKT pathway through the *zic4* enhancer induces brain tumour development

To generate a brain tumour model, we used the Gal4-UAS system to induce expression of different oncogenes under the *UAS* promoter in the driver line *Et(zic4:GAL4TA4,UAS:mCherry)_hmz5_* (Distel et al., 2009), henceforth referred to as zic:Gal4. This line expresses the codon-optimised version of the transcription factor *Gal4* under control of the *zic4* enhancer in the proliferating domains of the developing central nervous system (Fig. S1A-C′), which is visualised through mCherry expression. zic:Gal4 is also expressed in the adult brains (Fig. S1C,C′) as documented previously for the endogenous *zic4* gene (Aruga, 2004; Grinberg and Millen, 2005). We used different *UAS*-driven oncogenes, some activating the EGFR/RAS/ERK/AKT pathway, already reported to generate neoplasia in the zebrafish brain [*GFP-KRAS^V12^* (Ju et al., 2015), *AKT* (Jung et al., 2013)], others known to be oncogenic in human brain [*GFP-EGFR_transcript variant III (vIII)_* (Liu et al., 2005), also represented by *Xmrk*, the oncogenic version of the *EGFR* in *Xiphoporus*, and *BRAF^E600^* (Penman et al., 2015)] (see Table S1 for list and full names of constructs). All these oncogenes induced tumour formation (Fig. S2A-D, Table S2) and all but *AKT* induced ERK phosphorylation (Fig. S2E), with *GFP-HRAS^V1^^2^* exhibiting the strongest effect.

**Fig. 1.**
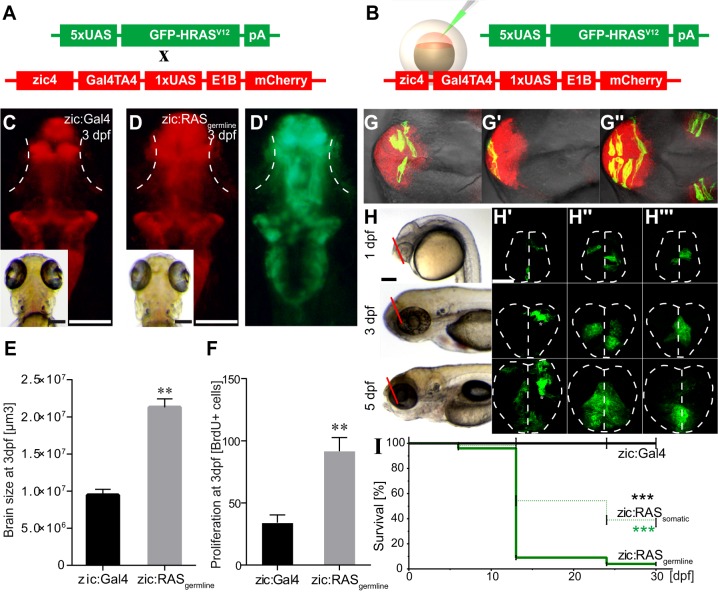
**Oncogenic RAS induces proliferation of neural progenitor cells, clonal expansion and reduced survival.** (A,B) Schematic representation of tumour induction through (A) the cross between zebrafish lines carrying the indicated transgenes or (B) the injection of the oncogenic construct (green) into zic:Gal4 embryos to express *UAS:GFP-HRAS^V12^* specifically in the brain. (C,D) Dorsal view of representative 3 dpf images of larvae showing the telencephalon in a control larva (C, zic:Gal4, mCherry expression) compared with that of an oncogenic larva (D, zic:RAS_germline_, mCherry expression; D′, zic:RAS_germline_, *UAS:GFP-HRAS^V12^* expression), white dotted lines mark the eyes. (E) Quantification of brain size reveals a doubling in size of the zic:RAS_germline_-expressing tissue. (F) Counting of BrdU-positive cells in the telencephalon of 3 dpf larvae reveals doubling in the number of proliferating cells in zic:RAS_germline_ versus zic:Gal4 controls. (G-G″) Dorsal view of three 1 dpf zic:RAS_somatic_ larvae showing individual clones expressing *UAS:GFP-HRAS^V12^*. (H-H″′) Lateral bright-field and coronal confocal images of the telencephalon of three live zic:RAS_somatic_ larvae (plane of focus indicated by red lines in H) at 1 dpf, 3 dpf and 5 dpf revealing clonal expansion of oncogene-expressing cells in the same larvae from 1 to 5 dpf. White dotted lines mark the outline of the brain. (I) Survival curve of zic:RAS_somatic_ larvae (green dashed line; *n*=166) compared with zic:Gal4 controls (black line, black asterisk; *n* =105) and zic:RAS_germline_ larvae (green solid line, green asterisk; *n* =255). Data are represented as mean±s.d. **P<0.03, ***P<0.001. Scale bars: 500 µm in C,D; 500 µm in H; 50 µm in H′.

To analyse the effect of activated RAS on tumour development, we generated both germline and somatic *UAS:GFP-HRAS^V12^­*-expressing animals. To induce germline *UAS:GFP-HRAS^V12^* expression (hereafter zic:RAS_germline_; Fig. 1A), we crossed the line zic:Gal4 to the line *tg(UAS:eGFP-HRASv12)_io006_* (Santoriello et al., 2010) (hereafter UAS:RAS). To induce somatic *UAS:GFP-HRAS^V12^* expression (hereafter zic:RAS_somatic_; Fig. 1B), embryos of the line zic:Gal4 were injected at the one-cell stage with the plasmid UAS:GFP-HRAS^V12^. F0 embryos expressing the transgene were identified by GFP expression and used for further analysis.

Both approaches induced early but different effects. The germline approach affected all *zic4*^+^ cells in the brain (Fig. 1C-D′) and already at 3 days post-fertilisation (dpf) led to a constant 2.3-fold increase in brain size (Fig. 1E), which was accompanied by a similar increase in the number of proliferating cells (mean±s.d. 91.6±11.0, *n*=5 in zic:RAS versus 34.1±6.2, *n*=5 in zic:Gal4 larvae) as determined through BrdU staining (2.7-fold increase; Fig. 1F). The somatic approach, by contrast, led to random oncogene expression in the *zic4*^+^ cell population, affecting single cells in different numbers and localisation (Fig. 1G-G″). Hence, it allowed for detailed time-lapse analysis of the affected cells, which revealed their clonal expansion in larvae (Fig. 1H-H″′). Even though both approaches led to tumour formation, the difference between them is mostly reflected in their survival, with the germline approach enabling only 4±1.2% to survive the first month whereas the somatic approach allowed 36.9±5.9% to survive (Fig. 1I).

These results indicate that germline expression of *UAS:GFP-HRAS^V12^* in the brain of developing zebrafish induces highly reproducible effects, enabling the possible application of this model in screening approaches. The somatic expression of *UAS:GFP-HRAS^V12^* is instead ideal for single-cell analysis as well as investigation of mechanisms of clonal expansion and oncogenesis up to later stages.

### Expression of the oncogene *GFP-HRAS^V12^* induces brain tumours and/or heterotopia

**Fig. 2.**
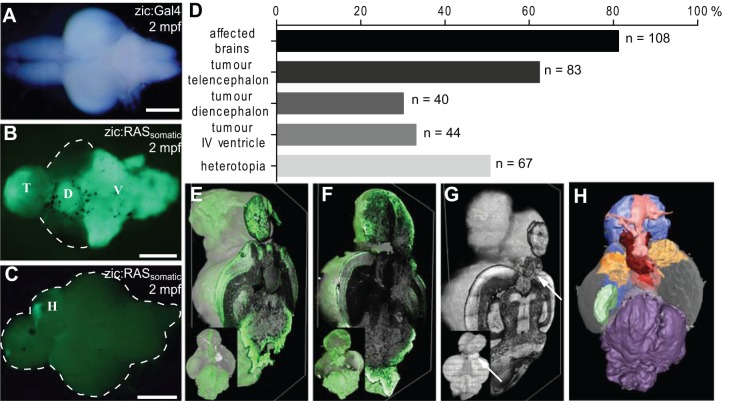
**Somatic expression of oncogenic RAS induces tumour development and heterotopia.** (A-C) Fluorescence images of 2 months post-fertilisation (mpf) zebrafish brains of zic:Gal4 (A) and zic:RAS_somatic_ (B,C) fish showing (A) a control brain, (B) tumours in the telencephalon (T), diencephalon (D) and IVth ventricle region (V), and (C) heterotopia (H). (D) Graph representing the frequency of different lesions resulting from induction of *UAS:GFP-HRAS^V12^* expression (*n*=134, of which T=83, D=40, V=44, H=67). (E-G) Volume rendering of 3D reconstructions of two brains with tumours (E,F, green areas) and a brain with heterotopia (G, white arrow) shown as dorsal view (inset) and one sagittal section (large image). (H) 3D reconstruction and volume rendering of a brain showing different tumour expansions (colour coded according to histological and anatomical features and GFP expression). Scale bars: 2 mm in A-C.

Next, brains of juvenile and adult fish (1-14 months) were resected and whole brains imaged for bright field and fluorescence. The observations unravelled the development of abnormal brain structures that could be grouped into malformations with and (mostly) without GFP expression (Fig. 2A-C). Both types of malformations occurred with the germline and with the somatic approach and were often found in the same brain (Table S2), but never in control-injected fish (data not shown). Specifically, the analysis of 134 brains of zic:RAS_somatic_ fish revealed that 81.2% of the brains developed GFP^+^ malformations, appearing most frequently in the telencephalon (62.4%), in the IVth ventricle (33.1%) and in the diencephalon (30.1%). However, 50.4% of the fish developed malformations, which were mostly GFP-negative (i.e. with only a few GFP^+^ cells, which for brevity we call GFP^−^), and both types of malformations could be present in the same brain (Fig. 2D). 3D reconstruction allowed analysis of the infiltrative nature of GFP-expressing lesions (Fig. 2E,F) whereas GFP^−^ malformations appeared as sharply circumscribed structures without penetration into deeper layers (Fig. 2G, white arrow). Further, the 3D reconstruction allowed us to predict that several clones contributed to the large GFP^+^ malformations, which we interpreted as cancerous growths based on the analysis reported in the following paragraphs (Fig. 2H).

### Specific immunohistochemical signatures establish that *GFP-HRAS^V12^*-positive lesions are tumours and show persistent activation of MAPK/ERK signalling

The relation between developmental brain lesions and glioma has been the focus of a number of recent reports (Hevner, 2015; Marin-Valencia et al., 2014; Reuss and von Deimling, 2009), raising the question of whether developmental brain growth disorders (including focal dysplasia and heterotopia; Aronica and Crino, 2014; Hevner, 2015) might be the result of halted oncogenic events taking place during development and/or provide a substrate for brain tumour development. In our model, the same oncogenes lead to two types of lesions, one resembling cancer and the other resembling heterotopia, thus providing an opportunity to study possible co-factors that might induce benign developmental lesions instead of tumours. The different nature of these lesions was assessed through H&E staining by an expert neuropathologist (P.L.P.), who also recognised peculiar features associated with tumours developing in the different areas of the brain. These features ranged from embryonal to more differentiated histopathological features, suggesting that these zebrafish brain tumours might resemble different histological subtypes of central nervous system tumours (Louis et al., 2016). To further clarify these differences and understand why in some instances the oncogene *HRAS^V12^* expressed in brain progenitor cells induced tumour development and in other cases produced only heterotopias, we investigated the expression of different markers by immunofluorescence: BrdU uptake for proliferation, GFAP for glial cells, S100β for progenitor cells, HU-C for neurons and p-ERK for MAPK activity.

**Fig. 3.**
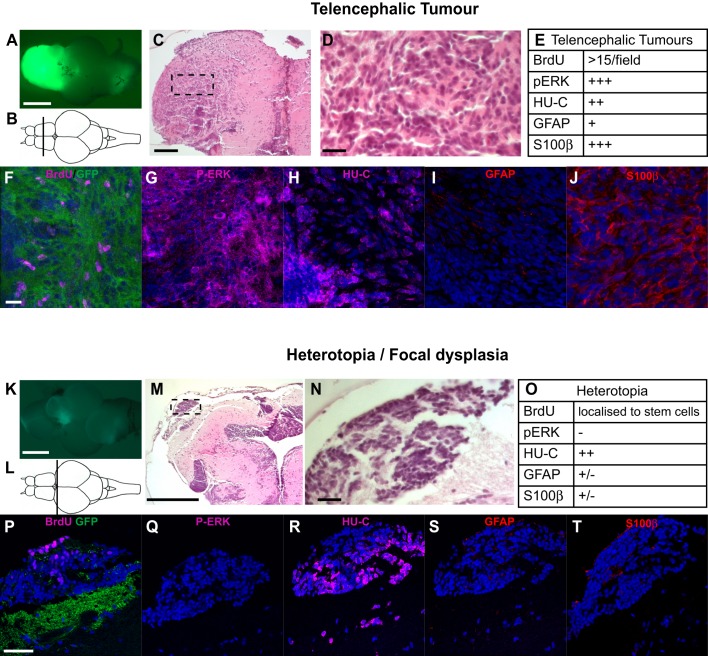
**Histological and immunological appearance of telencephalic tumour and heterotopia.** (A) Representative telencephalic tumour in zic:RAS_somatic_ fish. (B) Schematic drawing, indicating the position of the sections shown in F-J. (C) H&E-stained section, boxed area indicates enlargement shown in D. (E) Summary of the immunohistochemical observations related to telencephalic tumours. (F-J) Immunostaining of telencephalic tumour sections stained as indicated. DAPI as counterstaining is in blue. (K) Representative heterotopia in zic:RAS_somatic_ fish. (L) Schematic drawing, indicating the position of the sections shown in P-T. (M) H&E-stained section, boxed area indicates enlargement shown in N. (O) Summary of the immunohistochemical observations related to heterotopia. (P-T) Immunostaining of telencephalic tumour sections stained as indicated. DAPI as counterstaining is in blue. Scale bars: 2 mm in A,K; 200 µm in C,M; 25 µm in D,F-J,N,P-T.

The pattern of staining for these markers was disrupted in tumours and in heterotopias in different ways. Specifically, in the telencephalon, tumours (present in 62.4% of the injected fish, ‘T’ in Fig. 2B) appeared as diffusely infiltrating malignant masses (Fig. 3A-C) showing strong cellular heterogeneity (Fig. 3D) and a high proliferation index (Fig. 3E,F). Besides proliferation, we assessed p-ERK levels, number of HU-C^+^ and S100β^+^ cells and GFAP staining. A summary of the stainings present in GFP^+^ tumours arising in different brain regions is shown in Fig. 3F-J and Fig. S3.

GFP^−^ heterotopias (present in 50.4% of the injected fish; Fig. 2C) were defined as ectopically localised groups of cells lacking or with only a mild atypia and basically reproducing normal neural cell types but in the wrong location and/or in a larger number, reminiscent of a lack of maturation or migration and/or prolonged proliferation. They were easily visualised in the optic tectum (Fig. 3K-T), thanks to its layered structure, but also occurred in the telencephalon (Fig. S4A-A′) and in the cerebellum (Fig. S4B-B′). p-ERK staining in the heterotopias was absent (Fig. 3Q), whereas expression of HU-C, GFAP and S100β (Fig. 3R-T) was similar to that in the adjacent periventricular grey zone, from which the optic tectum heterotopias seem to originate. This suggested that normal developmental processes in these lesions were not subverted like in tumours, but just delayed or mis-localised. Most notably, *UAS:GFP-HRAS^V12^* expression (visualised through GFP fluorescence of the transgene) was barely detectable and no increase of ERK phosphorylation was detected in these lesions, suggesting that either the oncogene was switched off after initial expression, or that *UAS:GFP-HRAS^V12^*-expressing cells, which might have initiated abnormal migration and/or proliferation in the heterotopia, were eliminated so that at the time of the analysis, heterotopias were represented only by scar-like lesions.

In conclusion, the immunophenotype of these lesions revealed profound differences between heterotopias and tumours, despite their common origin from somatic expression of oncogenes. Inter-tumour variability was found (Fig. 3; Fig. S3), associated to the different areas of origin of the tumours.

### Analysis of global RNA expression established that brain tumours resemble GBMs of the mesenchymal signature, with a strong YAP component

To establish whether the zebrafish brain tumours developing in our models resemble a specific human molecular subtype, we performed transcriptome analysis by RNA sequencing (RNAseq) of three brains of zic:RAS_somatic_ fish, which carried tumour lesions in the telencephalon, diencephalon and IVth ventricle (Fig. S5A) and compared them with tumour-free, age-matched brains. Using hierarchical clustering on normalised gene expression, the samples clustered in two different groups according to their status (control or tumour; Fig. S5B). We performed an analysis of differential gene expression using DESeq2 (Love et al., 2014), and found 4194 genes differentially expressed (DE) (adjusted *P*-value <0.05) in brain tumour samples compared with controls. Of these, 2499 genes were upregulated and 1695 genes were downregulated (Fig. S5C,D).

**Fig. 4.**
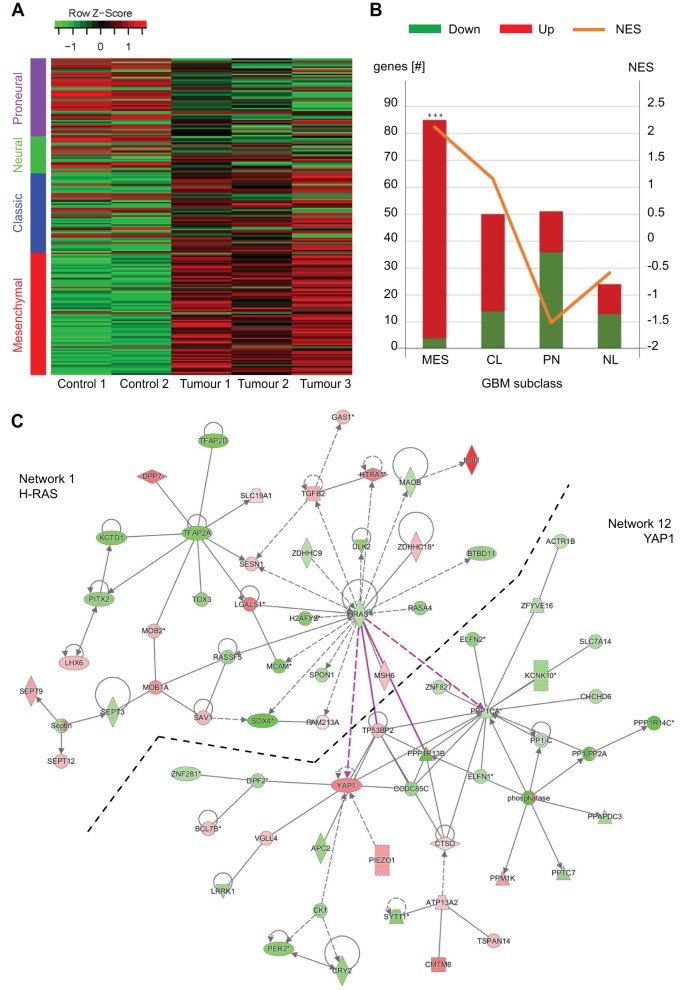
**The zebrafish brain tumours resemble mesenchymal subtypes of human GBMs.** (A) Heatmap comparing gene expression data of brains of zic:Gal4 (Control 1-2) and zic:RAS_somatic_ fish (Tumour 1-3) representing the normalised expression of the 248 significantly differentially expressed zebrafish orthologues of human GBM markers (adjusted *P*-value<0.05). After row scaling the zebrafish genes visually group into human GBM subclasses. (B) The Gene Set Enrichment Analysis (GSEA) on the orthologues of the GBM human markers shows that only the mesenchymal (MES) subclass is significantly enriched (FDR<0.001), associated with 82 zebrafish genes (orthologues of 85 GBM human markers). The classical (CL) subclass is associated with 53 zebrafish genes (50 GBM human markers), the proneural (PL) subclass with 52 zebrafish genes (51 GBM human markers) and the neural (NL) subclass with 27 zebrafish genes (24 GBM human markers). The orange line identifies the normalised enrichment score (NES). (C) IPA Network analysis suggests a close relation between HRAS and YAP in this tumour model as it connects HRAS (Network 1) with YAP (Network 12) via four connections (red lines).

Next, we evaluated whether the zebrafish brain tumours corresponded to a specific glioma subclass. Verhaak and colleagues identified 840 GBM markers useful to classify glioblastoma into four main subtypes (Verhaak et al., 2010). The same gene signature was later applied to low-grade brain tumours (Guan et al., 2014) and to mouse models of brain tumours (Henriquez et al., 2013). We first identified the zebrafish orthologues (Smedley et al., 2015) of the 840 human genes used by Verhaak et al. (2010). Owing to the presence of paralogs in the zebrafish genome (Howe et al., 2013), this resulted in a list of 1135 unique zebrafish Ensembl gene identifiers, which represented 91.31% of the 840 human GBM markers used by Verhaak et al. (2010) (Table S3). The four GBM subtypes were represented by similar numbers of orthologues in zebrafish (Fig. S5E). Using normalised expression data for the zebrafish orthologues of the human markers, we were able to classify the zebrafish brain tumours in one of the four GBM subtypes (Fig. 4A). Moreover, to further investigate the molecular features of our model, a gene set enrichment analysis (GSEA) was performed with the whole ranked list of significantly DE genes. Using this enrichment method, the mesenchymal subtype was the only significantly over-represented GBM subclass. We found 82 upregulated zebrafish genes with a normalised enrichment score (NES) of 2.12 and a nominal *P*-value <0.001, compared with the classical subclass (up=38, NES=1.16), the proneural subclass (down=38, NES=−0.59) and the neural subclass (down=16, NES=−1.51) (Fig. 4B; Table S3). Interestingly, among the 82 zebrafish orthologues of mesenchymal GBM markers found to be significantly upregulated in our model, five genes were related to YAP signalling: YAP1, WWTR1, TGFBR2, ITGB2 and IQGAP1 (Table S3). This observation prompted us to look at YAP­-related genes in the total list of 4194 DE genes. To do this, we first created a refined list of 39 zebrafish orthologues of human genes related to YAP signalling, based on literature (Anakk et al., 2013; Kodaka and Hata, 2015; Lim et al., 2014; Mo et al., 2014; Piccolo et al., 2014). Of them, 23 are significantly differentially expressed in the zebrafish brain tumours (adjusted *P*-value <0.05; Table S4), which confirmed the mesenchymal nature of our tumour model as YAP and TAZ signalling has been shown to be highly related to the mesenchymal subtype of GBM (Bhat et al., 2011; Orr et al., 2011).

To further investigate the importance of the role of mesenchymal markers, and in particular YAP, we used QIAGEN Ingenuity Pathway Analysis (IPA), which allowed us to identify possible upstream regulators, pathways involved and networks established by the DE genes in the zebrafish tumour model.

Of the 33,737 uploaded Ensembl zebrafish IDs, IPA mapped 11,896 to human IDs (21,841 left unmapped). IPA was able to assess the nature of the model based on the DE genes by returning ‘Cancer/Neoplasia’ as the most prominent Disease/Function in the dataset (Fig. S6A). Additionally, among the deregulated pathways, IPA identified cancer-related ones such as Gαq signalling, ERK/MAPK signalling, PI3K/AKT signalling and Hippo signalling (Fig. S6B, Table S5). Further, IPA ranks HRAS among the 15 most significant upstream regulators with a *P*-value of 3.03e–18. To understand which signalling molecules are responsible for the biological effects in our model, we performed a ‘Regulator Effects Network’ analysis. In this analysis, IPA connects upstream regulators via their target genes to known phenotypic and functional downstream effects. The generated networks are then ranked by the consistency score that is directly proportional to the number of consistent and inconsistent paths and indirectly proportional to the network size. The Regulatory Effects Network with by far the highest consistency score is shown in Fig. S6C. This network contains six regulators; of those, YAP had the highest interconnectivity, i.e. the highest number of relationships with other genes. Moreover, YAP is the only of the six regulators that has a high *P*-value in the IPA network analysis (Table S6). In this analysis the network headed by HRAS ranked first, whereas the YAP network ranked twelfth. Finally, the two networks (HRAS and YAP) are highly connected, as shown in Fig. 4C.

Taken together, these results suggest that YAP is an important regulator in this tumour model and indicate that tumours developing in this model have a mesenchymal gene signature, associated in humans with the most aggressive malignant glioma subtype. A YAP network based on the Ingenuity Pathway Knowledge Database, integrating our RNAseq expression data, is shown in Fig. S6D.

### YAP signalling is absent in heterotopia and expression of active YAP promotes development of aggressive brain tumours

**Fig. 5.**
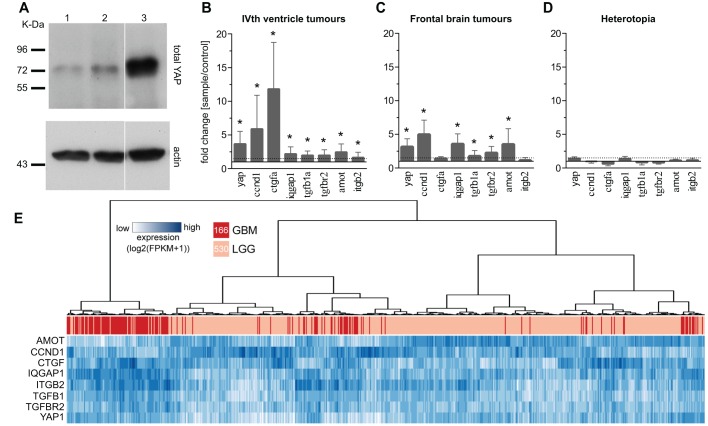
**A simple YAP signature distinguishes tumours from benign lesions.** (A) Western blot analysis shows increased YAP in tumour versus controls and heterotopia [1=control brain; 2=heterotopia; 3=tumour (*UAS:GFP-HRAS^V12^_germline_*]. (B-D) Expression of eight YAP target genes showing upregulation in tumours of the (B) IVth ventricle (*n*=6) and (C) frontal brain (*n*=6) and no upregulation in (D) heterotopia (*n*=5) when compared with control brains. Data are represented as mean±s.d. *P<0.05. (E) Hierarchical clustering of the gene expression data of the eight-gene YAP signature on 166 GBM (dark pink) and 530 LGG (light pink) samples using (1–Pearson's correlation) as distance measure. Gene expression is reported as fragments per kilobase of transcript per million fragments mapped (FPKM).

After showing the activation of YAP signalling in our model we next investigated the role of YAP activation on tumour formation in this model. To determine the activity of YAP in *UAS:GFP-HRAS^V12^*-induced tumours we detected the expression levels of YAP through western blot analysis and found a strong increase in total YAP expression in tumours compared with controls and brains with heterotopia (Fig. 5A). Further, we found an increase in YAP target gene expression using qPCR on 22 genes. These data compared well with the next-generation sequencing data on the same genes, of which 50% (11 genes) were similarly upregulated, 9% (2) were strongly upregulated and 41% (9) were not significantly altered (Fig S7A,B). We chose the eight most differentially expressed genes (*yap*, *ccnd1*, *ctgfa*, *iqgap1*, *tgfb1a*, *tgfbr2*, *amot*, *itgb2*) and tested this signature on different tumour types and heterotopia that showed overexpression of all eight genes in tumours of the IVth ventricle and of six genes in telencephalic tumours, but no overexpression in heterotopia (Fig. 5B-D). This suggests that YAP target gene expression differentiates tumours from heterotopia and that some YAP targets (*ctgfa* and *itgb2*) might be tissue- or tumour-specific.

To further confirm that the eight-gene YAP signature might be a useful molecular diagnostic tool, we analysed human tumour RNAseq data of 166 GBM and 530 brain lower grade glioma (LGG) generated by TCGA Research Network (http://cancergenome.nih.gov/). Unsupervised hierarchical clustering (Fig. 5E) demonstrates highly significant segregation of GBMs and LGGs (*P*<10^–45^, odds ratio=28, Fisher exact test) supporting that YAP activation is a hallmark of malignant brain cancer and suggesting that this eight-gene signature might provide sufficient information to distinguish high-grade from low-grade gliomas.

**Fig. 6.**
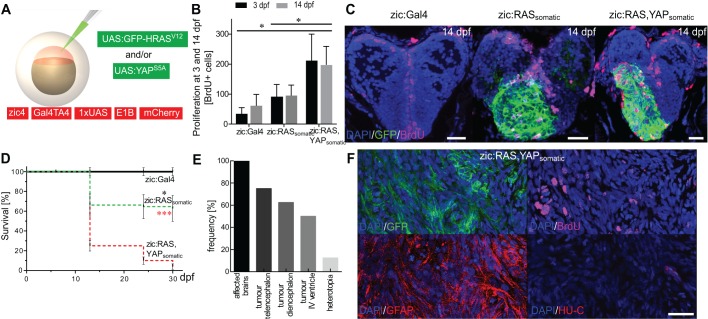
**YAP activation promotes tumour growth.** (A) Schematic representation of tumour induction through the co-injection of oncogenic constructs (green) into zic:Gal4 embryos. (B) Quantification of BrdU-positive cells in the telencephalon of 3 and 14 dpf juveniles reveals doubling in the number of proliferating cells in zic:RAS,YAP_somatic_ fish compared with zic:RAS_somatic_ fish and zic:Gal4 control fish. (C) Confocal image of 14 dpf fish showing BrdU expression (magenta) in zic:Gal4, zic:RAS_somatic_ and zic:RAS,YAP_somatic_. (D) Survival rate of zic:RAS_somatic_ (green dashed line; *n*=166) compared with zic:Gal4 (black solid line, black asterisk; *n*=105) and zic:RAS,YAP_somatic_ (red dashed line, red asterisks; *n*=100). (E) Graph representing the frequency of different lesions resulting from zic:RAS,YAP_somatic_ expression as determined by stereomicroscopic analysis with tumours in telencephalon (T), diencephalon (D) and IVth ventricle (V), and heterotopia (H). (F) Immunostaining for GFP, BrdU, GFAP and HU-C of zic:RAS,YAP_somatic_ fish. Data are represented as mean±s.d. *P<0.05; ***P<0.001. Scale bars: 20 µm in C,F.

Further, the effect of YAP activation in *UAS:GFP-HRAS^V12^*-induced tumours was investigated through expression of dominant­-active *YAP* (*YAP^S5A^*) under control of the *UAS* promoter. Somatic expression of *UAS:YAP^S5A^* alone (zic:YAP_somatic_) induced development of brain tumours (Fig. S8A,B) with YAP target gene expression (Fig. S8C), mixed cell populations (Fig. S8D) and reduced survival comparable with zic:RAS_somatic_ (Fig. S8E). By contrast, somatic co-expression of *UAS:GFP-HRAS^V12^* and *UAS:YAP^S5A^* (zic:RAS,YAP_somatic_; Fig. 6A) promoted tumour development earlier than in zic:RAS_somatic_ (2 weeks, data not shown), and increased proliferation at 3 dpf and 14 dpf (Fig. 6B,C) compared with zic:RAS_somatic_ larvae.

Somatic co-expression of both oncogenes was nearly incompatible with survival, allowing only 4±2.7% to survive the first month (Fig. 6D). However, the few survivors revealed a remarkable increase in the number of fish developing tumours of up to 100% and a sharp drop in heterotopia formation down to only 10% (Fig. 6E). Additionally, *UAS:YAP^S5A^* promoted aggressiveness of the developing tumours characterised by strong proliferation and fast dedifferentiation, as determined by strong staining for GFAP and nearly complete lack of HU-C staining (Fig. 6F).

Thus, YAP activation not only co-operates with *UAS:GFP-HRAS^V12^* in promoting tumour development, but might also function to overcome mechanisms halting tumour development when oncogenes are accidentally expressed in somatic cells during brain development.

## DISCUSSION

The progression of pre-malignant developmental lesions to tumours has been proved for several tissues such as colon (Macrae et al., 2009) and pancreas (Aguirre et al., 2003). In the brain, the relation between focal brain developmental disorders and brain cancer has been the subject of several studies aiming at establishing whether a common genetic and developmental origin for these disorders exists in cases with clear evidence of progression (Aronica and Crino, 2014; Guerrini and Dobyns, 2014; Hevner, 2015). However, for the majority of brain tumours, evidence of a developmental origin of the somatic mutations driving cancer is difficult to obtain. In this study, we generated a model of progressive brain tumour development where the same genetic drivers can give rise to cancer and heterotopia, and identified the signalling pathway that, once activated, promotes tumour development at the expense of heterotopia. Our model suggests that somatic embryonic mutations activating MAPK/ERK signalling can drive both malformation of brain development and brain tumours, proving that upregulation of YAP signalling is necessary for tumour development.

Somatic mutations in pro-oncogenic factors occurring during development start to be recognised as an important determinant of congenital brain malformation and neurodevelopmental disorders spanning from Proteus syndrome to neurofibromatosis type I (reviewed in Poduri et al., 2013). By contrast, somatic pro-oncogenic mutations occurring in post-developmental stages are often associated with cancer. Several studies have suggested a possible progression between neuro-developmental lesions and brain cancer, especially when the activating mutations induce MAPK/ERK signalling (Hevner, 2015). If a progression is possible from non-cancerous neuro-developmental lesions caused by activating mutations in a pro-oncogenic pathway and brain cancer, then an important topic for future research is to identify the mechanisms that restrain affected cells from developing cancer during development, and lead to reactivation of a dormant oncogenic program in case of progression to cancer. However, until now no animal model has been described to allow the investigation of the link between heterotopia and tumour formation.

In our study we have used different oncogenes to generate a model of brain growth disorders in zebrafish. This model shows that RAS/MAPK signalling can simultaneously induce both heterotopia and aggressive brain tumours and that the persistence of the signal differentiates brain tumours from benign developmental lesions. The reduction or absence of *GFP-HRAS^V12^* expression in the heterotopia suggests that this could result from cells expressing *UAS:GFP-HRAS^V12^* turning off activated RAS after initial expression or undergoing cell death after influencing the ectopic migration of their neighbouring cells. Further studies are needed to clarify this point. In the second part of our study we scrutinised the transcriptional programs activated in those lesions that progress to cancer. We focused on the factors that seem to be responsible for maintaining ERK signalling in some neural cells, which will then form tumours, or shut it down in others, which will subsequently develop benign heterotopias. We found that the gene expression profile of RAS/MAPK tumours resembles the mesenchymal GBM signature, reported by Verhaak et al. (2010), which underlines their comparability to the human disease. Additionally, the zebrafish brain tumours expressed a strong YAP component. YAP (or Yap1) is a transcription co-factor, shut off by the Hippo pathway, which controls organ size during development (reviewed in Meng et al., 2016). YAP is part of a classic phosphorylation cascade and is activated through different mechanisms to promote growth and migration in cancer (reviewed in Yu et al., 2015).

For example, pancreatic adenocarcinoma was shown to have a strong YAP component, as KRAS-induced acinar-to-ductal metaplasia depends on YAP expression for progression to malignant ductal adenocarcinoma (Zhang et al., 2014). Moreover, cancer cells can use YAP to compensate for loss of mutant KRAS as shown in cell lines and mouse models of pancreatic cancer (Kapoor et al., 2014; Shao et al., 2014). In high-grade gliomas, YAP has been shown to be activated especially in aggressive tumour types and its expression tends to correlate with low survival rates (Bhat et al., 2011; Orr et al., 2011). However, the molecular mechanisms behind this correlation are unknown. Analysis of medulloblastoma suggests that upstream regulators of the Hippo pathway control the activation of YAP in brain cancer. Indeed, inhibition of NF2 (encoded by *MERLIN*) induces nuclear localisation and activation of YAP, which can be rescued by YAP inhibition (Piccolo et al., 2014). Interestingly, the inheritable brain dysplasia Van Maldergem syndrome (VMS; MIM601390) results from mutations of the Hippo upstream regulators *Dchs1* and *Fat4*, and the phenotype in relevant mouse models can be rescued by YAP inhibition (Cappello et al., 2013).

YAP activity can also be regulated through other mechanisms including interaction with cellular compartments such as the actin cytoskeleton. The actin cytoskeleton has been shown to be crucial for YAP nuclear localisation (Shao et al., 2014), sometimes in cooperation, but more often independently, of the Hippo pathway (Aragona et al., 2013). F-actin translates the mechanical signals from the extracellular matrix to the cell. As a component of the tumour microenvironment, the extracellular matrix has a significant impact on the development, progression, and therapy response in tumours (Giussani et al., 2015; Lu et al., 2012). F-actin can relay its effect on YAP through several mechanisms such as G-protein-coupled receptors (GPCRs), which are known to combine the actin cytoskeleton with several signalling pathways (reviewed in Regue et al., 2013), or IQGAP1, a scaffold protein known to regulate the F-actin and microtubule network and shown to play a pivotal role in a bile acid-induced liver cancer through YAP (Anakk et al., 2013). Thus, YAP activation not only correlates with increased proliferation but might promote tumour progression through interactions with the tumour environment. However, the specific mechanism through which YAP translates physical inputs into cancer-promoting signals is still to be elucidated.

Our study shows an additional role of YAP in tumour development as expression of dominant-active YAP demonstrates its co-operation with oncogenic RAS in the induction of brain cancer instead of neuro-developmental lesions. The mechanisms through which oncogenic RAS induces YAP activation only in some lesions and after some time (3 weeks) from its initial expression are currently unknown, but might involve a downregulation of members of the ubiquitin ligase complex that target YAP for degradation (SOCS5/6; Hong et al., 2014), or F-actin through GPCRs or IQGAP1. Further studies will clarify this point.

In contrast to currently available rodent models on brain dysplasia or brain tumours, this zebrafish glioma model provides the advantage of simultaneous development of tumours and heterotopia in a nearly equal ratio, induced by the same oncogene, which enables the analysis of the mechanisms that control the fate decision and the requirements for progression. Moreover, the model enables time-dependent investigation of tumour progression in a living vertebrate on a single­-cell level. Whereas development of brain tumours has also been investigated in other zebrafish models (Ju et al., 2015, 2014; Solin et al., 2015), the model described here provides the advantage of highly frequent development of both heterotopias and neoplastic malignant lesions in more than 80% of the specimens and very early onset of oncogenic processes, which not only shortens observation times but also allows for efficient screening of therapeutic agents, using prevention of tumour development and tumour progression as read-outs. Further analysis of the model developed in this study can provide an understanding of the mechanisms that promote the progression of benign lesions to malignant tumours and a convenient assay for testing inhibitory treatments that could prevent malignant transformation of developmental brain lesions.

## MATERIALS AND METHODS

### Animal housing and line generation

All fish lines were raised and maintained under standard conditions (Westerfield, 2000). Fish with mosaic somatic plasmid expression were generated by co-injection of 0.25 ng/µl DNA (see Table S1) and 0.25 ng/µl mRNA encoding Tol2 transposase into the cell of one-cell-stage embryos. Embryos were kept at 28.5°C in E3 solution and 0.003% PTU (1-phenyl-2-thiourea, Sigma Aldrich, Germany) was added to the media at 24 hpf to reduce pigmentation. For line generation, carriers were selected by fluorescence and outcrossed to wild-type strains as adults to generate F0. At least two different F0 per line were analysed, to identify potential insertion effects. No differences between alleles of the same transgenic lines were found, therefore we selected only one of them for further studies.

All animal experiments were carried out under EU regulations for animal experimentation. The project was approved by the Government of Baden-Württemberg, Regierungspräsidium Karlsruhe, Germany under Aktenzeichen 35-9185.81/G-41/14.

### Survival curve

For survival analysis fish were housed in groups of ≤30 and survivors were counted at 0 dpf, 6 dpf, 13 dpf, 24 dpf and 30 dpf. In this and all other comparative analyses, zic:Gal4 fishes and their brains were used as controls. The length of these intervals was decided after pilot observations that showed that the majority of the zic:RAS larvae died at around 10-12 dpf. For each curve at least three repeats were performed. The total numbers of animals used for the survival curve was 105 (zic:Gal4), 255 (zic:RAS_germline_), 166 (zic:RAS_somatic_), 100 (zic:RAS,YAP_somatic_).

### Live imaging of larvae

For live imaging, larvae were anaesthetised with 0.02% tricaine methanesulfonate (tricaine, Sigma Aldrich) in E3, embedded in 1% low-melting-point (LM) agarose in E3 and imaged with a stereo microscope (Leica MZTL III), Leica DFC42 digital camera, LAS V4.5 software or with a confocal microscope (Leica DMI 4000B) and LAS X software (Leica Microsystems, Germany). For repeated imaging, larvae were removed from agarose after each imaging, and housed in 24-well plates as single larvae in E3 mixed with PTU at 28.5°C until the next imaging session.

### Brain size quantification

At 3 dpf zic:Gal4 (*n*=12) and zic:RAS_germline_ (*n*=16) larvae were anesthetised with 0.02% tricaine in E3, embedded in 1% LM agarose in E3 and the whole brain imaged from dorsal to ventral using a confocal microscope (Leica DMI4000B) under the following settings: objective ACS APO 10.0×0.30 DRY; zoom 1.0; z-slice 2 µm; resolution 512×512. Using MATLAB (MathWorks) each stack of images was assigned a manual threshold and a manual region of interest covering the rostral brain until mid-brain boundary. Using a manual threshold, the RGB images were binarised, followed by a dilation (r=5), hole filling, erosion (r=5) and opening (r=10). The extracted images were cropped using a binary region of interest. The resulting images were used to build a 3D-structure and the volume was quantified (number of voxels×volume per voxel).

### BrdU, H&E and immunostaining

Fish were killed by anaesthetic overdose (0.04% tricaine) and brains dissected under a stereomicroscope. All samples were fixed in 4% PFA for 24 h before paraffin embedding. For proliferation analysis fish were incubated in 10 µM bromodeoxyuridin (BrdU, Sigma Aldrich) in E3 24 h prior to sample collection. In 10 larvae per group, total number of BrdU^+^ cells were counted in coronal sections of the telencephalic areas; in adults BrdU^+^ cells in tumours, heterotopias or in similar regions of control brains were counted in 2-3 sections of five different samples (referring to an area of 0.5 mm^2^) given as fraction of total cell number (DAPI^+^ cells) in that field.

For histological analysis 10 µm serial sections were stained with haematoxylin and eosin (H&E). Images were acquired using a light microscope (Zeiss Axioscope), AxioCam HRc camera and AxioVision SE64 Rel. 4.9.1 software (Zeiss).

For immunohistochemical analysis sections were demasked with a citrate buffer antigen retrieval protocol (Brown and Chirala, 1995) and stained with primary antibodies against glial fibrillary acid protein (GFAP, 1:1000, Dako, Germany, Z0334), S100β (1:1000, Dako, Z0311), HU-C (1:200, Life Technologies, USA, A21271), phospho-ERK (p-ERK, 1:200, Cell Signaling Technologies, USA, 9101S), green fluorescent protein (GFP, mouse 1:500, Millipore, Germany, MAB3580 or rabbit 1:1000, Life Technologies, A11122). For staining with the antibody against BrdU (1:500, Cell Signaling Technologies, 5292S) sections were additionally treated for 20 min with 2 N HCl. All sections were stained with fluorescently labelled secondary antibodies against rabbit or mouse immunoglobulins (1:200, Life Technologies, A11017, A11018, A11070, A11071, A21050, A21070). Images were acquired using a confocal microscope (Leica DMI 4000B) and LAS X software.

To obtain the summaries of immunostainings shown in Fig. 3E,O at least five different tumours per region or heterotopias were examined. Number of positive cells or percentage of positive area was evaluated in an area of ∼0.5 mm^2^ in three different sections per tumour or heterotopia. The symbols are representative of these counts: less than 5% positive cells/area, +/–; between 5 and 25%, +; between 25 and 50%, ++; over 50%, +++.

### RNA analysis

All fish were killed by anaesthetic overdose (0.04% tricaine) and brains of juveniles and adults dissected under a stereomicroscope. Larvae and tissue samples for RNA extraction were collected and lysed in trizol (Life Technologies) and total RNA extracted with the RNeasy Mini Kit (QIAGEN, Germany) following manufacturer's protocol. Samples for quantitative PCR (qPCR) analysis were additionally treated with RNA-Free DNase (QIAGEN) for 30 min at room temperature.

For gene expression analysis via qPCR RNA samples were transcribed to cDNA using the SuperScript^®^ ViloTM cDNA Synthesis Kit (Invitrogen, USA) and qPCR was performed using the Gotaq^®^ qPCR Master Mix (Promega, Germany) following manufacturer's protocol in the StepOnePlus Real-Time PCR System (Applied Biosystems, Germany), with the following setting: 95°C, 15 min; 40× (95°C, 15 s; 60°C, 30 s); 95°C, 15 s; 60°C, 1 min; melting curve 0.5°C per 15 s to 95°C. Data were analysed with the StepOne Software v2.3 (Thermo Fisher). For normal PCR cDNA samples were diluted 1:10 and 10 µl added to PCR mix (10.5 µl dH_2_O, 1 µl dNTPs (10 mM mix), 1 µl primer each, 0.5 µl GoTaq^®^ (Promega), 8 µl 5×buffer (provided by GoTaq^®^ kit) and amplified in the T100™ Thermal Cycler (BioRad, USA) [95°C, 5 min; 27× (95°C, 30 s; 60°C, 30 s; 72°C, 30 s) 72°C, 5 min; 12°C hold]. Primer pairs are listed in Table S8.

For next-generation sequencing, total RNA samples were extracted using the RNeasy Mini Kit (QIAGEN), and quality was assessed on RNA nanochips (Agilent Bioanalyser 2100, USA). The libraries were prepared from 1 µg RNA using the Illumina TrueSeq mRNA kit (Illumina, USA) according to the supplier's protocol. The size and the concentration of the libraries were determined with DNA-chip (Agilent Bioanalyser 2100). A normalised concentration of 8 pM of the libraries was loaded on one lane of a high throughput sequencing flowcell (Illumina) to generate the clusters, using a cBot (Illumina). The sequencing of the paired-end reads (2×50 nucleotides) was done using an Illumina HiSeq1500 with SBS v3 kits (Illumina). The cluster identification and the base calling were done using RTA v1.13 (Illumina) and the quality of the reads was assessed with CASAVA v1.8.1 (Illumina). The sequencing resulted in an average of 112 millions of reads per sample with, on average, 97% having a quality Phred score greater than 30. The quality of the raw sequencing data was assessed using fastx-toolkit (version 0.0.13) (http://hannonlab.cshl.edu/fastx_toolkit/index.html) and no pre-processing of the data was necessary. The alignment was done using TopHat2 (version 2.0.11) (Kim et al., 2013) against the assembly Zv9 Ensembl 75 of the *Danio rerio* genome with the parameters -r 180 --mate-sdt-dev 80 --b2-sensitive --no-novel-junction -a 5 -p 3 --library-type fr-unstranded. The raw gene expression was computed using HTSeq (version 0.5.3p3) (Anders et al., 2015) with --stranded=no --mode=union parameters. The raw sequencing data (fastq files) and the pre-processed data (count files) were submitted to the Gene Expression Omnibus database (http://www.ncbi.nlm.nih.gov/geo/query/acc.cgi?acc=GSE74754). The normalisation of the gene expression and the differential gene expression were both computed using DESeq2 (Love et al., 2014). At this step, the consistency of the biological replicates was tested using hierarchical clustering in a complete mode on Euclidean distances. One control replicate was discarded at this stage for the rest of the analysis. The significantly differentially expressed (DE) genes were selected based on an adjusted *P*-value of less than 0.05, using the Bonferroni multiple testing method. No cut-off was used on the log2 fold-change. To assess the role of GBM subtype markers on the zebrafish tumour model, the names of the 840 GBM subtype markers, published by Verhaak et al. (2010), were retrieved from TCGA data portal. For the rest of the analysis the markers for GBM subtype were kept but also markers not associated with a specific GBM subtype, labelled ‘non type-specific’ (Table S3). The zebrafish orthologues were then found using the Ensembl database and the BioMart portal (Smedley et al., 2015). A curation was applied using 30% as minimum cut-off for the gene sequence identity or 1 as orthology confidence score cut-off. The list of orthologues was then manually refined for highly important genes. A total of 1135 zebrafish orthologues were found owing to the presence of paralogues in the zebrafish genome. The four GBM subtypes were similarly represented (Fig. S5E). To investigate the involvement of markers of a specific GBM subtype, the pre-ranked algorithm of Gene Set Enrichment Analysis (GSEA) (Subramanian et al., 2005) software package developed by the Broad Institute was used. The significantly DE orthologues of the GBM subtype markers were ranked according to their log2 fold-change and then used for enrichment with GSEA for the four GBM subtype gene sets.

### Analysis of human gene expression data

Normalised gene expression data (RNASeqV2) of the signature genes for LGG (*n*=530) and GBM (*n*=166) were downloaded from cBIOPortal (http://www.cbioportal.org/) (Cerami et al., 2012) by selecting the study identifiers ‘lgg_tcga’ and ‘gbm_tcga’, respectively. The segregation of LGG and GBM samples was tested by Ward's hierarchical clustering, using (1–Pearson's correlation coefficient) as distance measure. Statistical significance of LGG and GBM segregation was estimated by two-sided Fisher exact test on the two main clusters.

### IPA analysis

To predict the effects of gene expression changes in the model QIAGEN's Ingenuity Pathway Analysis (IPA^®^, QIAGEN, USA, www.qiagen.com/ingenuity) was applied. The complete RNAseq dataset containing the quantitative expression values and corresponding adjusted *P*-values of all genes comparing zebrafish control brain and zebrafish brain tumour samples was uploaded to IPA and the cut-off for gene analysis set to 0.05. IPA automatically translated zebrafish gene IDs into human gene IDs. For analysis of ‘Disease or Function’ and ‘Upstream Regulator’ the default settings from IPA were applied.

As the RNAseq samples exclusively contained brain tissue the analysis of ‘Regulatory Effects Network’ was restricted accordingly by removing liver, kidney, lung, skeletal, cardiac and sensory organ effects from the analysis.

For the analysis of ‘Network’ the default settings from IPA were applied, which restricts the outcome to the 25 most significant networks. These are constructed between genes of the dataset according to the number of known interactions with other genes, assuming that the number of interactions correlates with the biological relevance of the gene product. The networks are sorted by ‘Score’ which reflects their interconnectivity.

### Western blot

Fish were killed by anaesthetic overdose (0.04% tricaine) and their brains dissected under a stereomicroscope. The samples were homogenised in sample buffer (5% glycerol, 1.7% SDS, 60 mM Tris HCl pH 6.8, 0.01% EDTA) containing protease inhibitors (cOmplete, Roche, Germany) and phosphatase inhibitors (phosphoStop, Roche). Equal amounts (20-50 mg) of the total extract were separated on 10% acrylamide gels and transferred to a PVDF membrane using Trans-Blot^®^ Turbo™ RTA Transfer Kit, PVDF (BioRad) and Trans-Blot^®^ Turbo™ Transfer System machine (BioRad). The membrane was blocked in 2% BSA and incubated with the following antibodies overnight: p-ERK (1:200, Cell Signaling Technologies, 9101S) and YAP (1:200, Cell Signaling Technologies, 4912). After washing, the membrane was incubated 1.5 h at room temperature with horseradish peroxidase-conjugated goat anti-mouse IgG (Dako, P0447) or goat anti-rabbit IgG (Dako, P0448), washed again and activated with Pierce^®^ ECL Western Blotting Substrate (Thermo Scientific, USA) system. For reuse, the membrane was treated with Restore™ Western Blot Stripping Buffer (Thermo Scientific) according to the supplier's protocol. For normalisation, antibodies against total ERK (1:200, Cell Signaling Technologies, 9102) or actin (1:5000, Neomarkers-Fremont, USA, ACTN05) were used on stripped membranes.

### Cloning

For the generation of transgenes expressing *UAS:BRAF^V600E^*, *UAS:Xmrk*, *UAS:EGFR _splice variant III_* (shortened in _vIII_), *UAS:Yap^S5A^* and *UAS:lifeact-GFP* we used different strategies. As a backbone (vector) we used pT2MUASMCS (a kind gift from Koichi Kawakami, National Genetic Institute, Mishima, Japan), which contains a Tol2-based flanking cassette for genomic integration, and five *UAS* repeats before the multiple cloning site. We used conventional cloning of blunt fragments in the *Eco*RV cloning site of the pT2MUASMCS vector, followed by 5′ and 3′ sequencing to check orientation and integrity of the insert. Inserts were GFP-tagged using gateway recombination with pEntry clones of the Tol2kit (Kwan et al., 2007) before being cloned into pT2MUASMCS. For *UAS*-controlled myristoylated AKT1 we used gateway recombination to clone a 5×UAS:Akt1:5×UAS:BFP construct into pDEST Tol2 CG2.

The plasmids containing the different oncogenes that were used as templates in Gateway cloning were kind gifts of the following labs: *BRAF^V600E^* (Liz Patton, MRC, Institute of Genetics and Molecular Medicine, Edinburgh, UK); *Xmrk* (Manfred Schartl, Department of Physiological Chemistry, University of Würzburg, Würzburg, Germany), pcDNA3 Myr HA Akt1 was a gift from William Sellers (Addgene plasmid #9008) (Ramaswamy et al., 1999), MSCV-XZ066-EGFRvIII was a gift from Alonzo Ross (Addgene plasmid #20737) (Li et al., 2009), *YAP^S5A^* (Sirio Dupont, Dipartimento di Medicina Molecolare, University of Padova, Padova, Italy). Lifeact-GFP was obtained from the authors of Riedl et al. (2008).

### 3D visualisation

#### CLARITY procedure

Whole-dissected adult brains were fixed in freshly prepared ice-cold methanol-free paraformaldehyde (PFA) 4% (w/v) in 0.01 M PBS (pH 7.4) overnight at 4°C. Samples were then infused in a pre-cooled (4°C) solution of freshly prepared hydrogel monomers [0.01 PBS, 0.25% VA-044 initiator (w/v), 5% dimethyl sulfoxide (v/v), 1% PFA (w/v), 4% acrylamide (w/v) and 0.0025% bis-acrylamide (w/v)] for 2 days at 4°C. After degassing the samples the hydrogel polymerisation was triggered by replacing atmospheric oxygen with nitrogen in a desiccation chamber for 3 h at 37°C. Samples were cleaned from superfluous hydrogel and transferred into embedding cassettes for lipid clearing. Passive lipid clearing was performed at 40°C for 8 days in clearing solution [8% SDS (w/v), 0.2 M boric acid, pH adjusted to 8.5] under gentle agitation. Subsequently the samples were thoroughly washed in 0.01 M PBS, tween 0.1% (w/v) (PBSt) at room temperature with gentle agitation for 2 days.

#### Immunostaining of clarified samples

CLARITY-processed brains were incubated in blocking solution [0.01 M PBS, 0.1% Tween 20 (v/v), 1% Triton X-100 (v/v), 10% dimethyl sulfoxide (v/v), 10% normal goat serum (v/a), 0.05 M glycine] overnight at 4°C. Subsequently samples were incubated in staining solution [0.01 M PBS, 0.1% Tween 20 (v/v), 0.1% Triton X-100 (v/v), 10% dimethyl sulfoxide (v/v), 2% normal goat serum (v/v), 0.05% azide (v/v)] with primary antibody (1:400, chicken anti-GFP, Avès Labs, USA, GFP-1020) for 7 days at room temperature under gentle agitation. After four washing steps in PBSt, samples were incubated in staining solution with secondary antibody (1:400, goat anti-chicken Alexa Fluor 488, Invitrogen, A-11039) for 7 days at room temperature. Samples were washed for 2 days in PBSt and stained with 1 µM DiIC18(3) solution (DiI Stain, Molecular Probes, USA).

#### Imaging in high refractive index solution

A fructose-based high refractive index medium (fruM) was prepared as follows: 70% fructose (w/v), 20% DMSO (w/v) in 0.002 M PBS, 0.005% sodium azide (w/v). The refractive index of the solution was adjusted to 1.4571 using a refractometer (Kruss).

In preparation for imaging the samples were incubated in 50% (v/v) fruM for 6 h and finally incubated in 100% fruM for at least 12 h. For imaging, samples were mounted in 1% (w/v) low-melting-point agarose and covered with fruM. Whole-mount brain fluorescence was recorded with a Leica TCS SP8 two-photon microscope. Fluorescence was excited using a mode locked Ti:Sapphire laser (Chameleon, Coherent) at 770 nm with the Leica HC FLUOTAR L 25×/1.00 IMM motCorr objective. Non-descanned detectors with 525/50 and 585/40 bandpass filters were used for data acquisition. As the specimens are significantly bigger than the field of view of the used objective tiled scanning with a voxel size of 0.9×0.9×1 µm or 1.7×1.7×1.7 µm was applied.

#### Image treatment and visualisation

In preparation for visualisation the image stacks were converted from their native 12 bit lif format to series of 8 bit pngs using CLAHE (Zuiderfeld, 1994) for ImageJ (Schneider et al., 2012) as implemented in Fiji (Schindelin et al., 2012). The implementation is described online (http://fiji.sc/Enhance_Local_Contrast_%28CLAHE%29). The parameters for CLAHE were empirically tested and set to a blocksize of 127, 256 bins and a slope of 3 (default values). When reducing the bit depth from 12 bit to 8 bit Fiji's CLAHE plugin enhances the contrast and intensity of the weak signals significantly while not over-saturating strong signals. By this method a significant contrast enhancement and data reduction can be achieved. Manual segmentation and 3D rendering was performed with amira (www.fei.com) using a combination of the ‘Segmentation Editor’, ‘Voltex’, ‘Volume Rendering’ and ‘Surface View’ modules.

### Statistics

For statistical analysis GraphPad Prism 6 was used applying unpaired Student's *t*-tests and Bonferroni correction. Values are given in mean±standard deviation (s.d.).
